# Multi-Parametric Magnetic Resonance Imaging-Based Radiomics Analysis of Cervical Cancer for Preoperative Prediction of Lymphovascular Space Invasion

**DOI:** 10.3389/fonc.2021.663370

**Published:** 2022-01-12

**Authors:** Gang Huang, Yaqiong Cui, Ping Wang, Jialiang Ren, Lili Wang, Yaqiong Ma, Yingmei Jia, Xiaomei Ma, Lianping Zhao

**Affiliations:** ^1^ Department of Radiology, Gansu Provincial Hospital, Lanzhou, China; ^2^ The First Clinical Medical College, Gansu University of Chinese Medicine, Lanzhou, China; ^3^ GE Healthcare China, Beijing, China

**Keywords:** uterine cervical neoplasms, radiomics, lymphovascular space invasion, magnetic resonance imaging, predictive value of tests

## Abstract

**Background:**

Detection of lymphovascular space invasion (LVSI) in early cervical cancer (CC) is challenging. To date, no standard clinical markers or screening tests have been used to detect LVSI preoperatively. Therefore, non-invasive risk stratification tools are highly desirable.

**Objective:**

To train and validate a multi-parametric magnetic resonance imaging (mpMRI)-based radiomics model to detect LVSI in patients with CC and investigate its potential as a complementary tool to enhance the efficiency of risk assessment strategies.

**Materials and Methods:**

The model was developed from the tumor volume of interest (VOI) of 125 patients with CC. A total of 1037 radiomics features obtained from conventional magnetic resonance imaging (MRI), including a small field-of-view (sFOV) high-resolution (HR)-T2-weighted MRI (T2WI), apparent diffusion coefficient (ADC), T2WI, fat-suppressed (FS)-T2WI, as well as axial and sagittal contrast-enhanced T1-weighted MRI (T1c). We conducted a radiomics-based characterization of each tumor region using pretreatment image data. Feature selection was performed using the least absolute shrinkage and selection operator method on the training set. The predictive performance was compared with single variates (clinical data and single-layer radiomics signatures) analyzed using a receiver operating characteristic (ROC) curve. Three-fold cross-validation performed 20 times was used to evaluate the accuracy of the trained classifiers and the stability of the selected features. The models were validated by using a validation set.

**Results:**

Feature selection extracted the six most important features (3 from sFOV HR-T2WI, 1 T2WI, 1 FS-T2WI, and 1 T1c) for model construction. The mpMRI-combined radiomics model (area under the curve [AUC]: 0.940) reached a significantly higher performance (better than the clinical parameters [AUC: 0.730]), including any single-layer model using sFOV HR-T2WI (AUC: 0.840), T2WI (AUC: 0.770), FS-T2WI (AUC: 0.710), ADC maps (AUC: 0.650), sagittal, and axial T1c values (AUC: 0.710, 0.680) in the validation set.

**Conclusion:**

Biomarkers using multi-parametric radiomics features derived from preoperative MR images could predict LVSI in patients with CC.

## Introduction

Cervical cancer (CC) is the fourth most frequently diagnosed cancer worldwide, with an increasing incidence rate, high mortality rate, and a younger age of onset (1). Lymphovascular space invasion (LVSI) refers to the presence of cancer cell clusters in the vascular and/or lymphatic lumen (2). LVSI increases the risk of lymph node metastases (LNM), which is an independent high-risk factor for tumor recurrence and influences treatment methods (3–5). Studies have shown that LVSI is associated with decreased survival rates in women with stage IA2 (5), IB, and II CC [defined according to the Federation International of Gynecology and Obstetrics (FIGO)] (3, 6). Satellite LVSIs also significantly increase the risk of death and recurrence (7). A definite preoperative distinction is of great clinical importance in the management of patients with CC. The LVSI status should be considered for a more accurate risk stratification (3). However, it is challenging to determine the decisive characteristics of LVSI in clinically relevant biomarkers due to intralesional heterogeneity, extra time delays, and high testing costs. A definitive diagnosis can only be made through a histopathological examination of the surgical specimens. However, pathological analysis is not universally applicable, and there is wide interobserver variability in determining LVSI (5). A reformative, non-invasive, real-time, and cost-efficient preoperative test method for risk identification will have a considerable clinical impact on women with CC.

As an emerging field of medical imaging, radiomics refers to the process of extracting and analyzing massive quantitative features from medical images to achieve the ultimate goal of prediction or prognosis (8). Based on sufficient spatial resolution, multi-parametric magnetic resonance imaging (mpMRI), including high-resolution T2-weighted MRI (HR-T2WI), diffusion-weighted imaging (DWI), and dynamic contrast enhancement (DCE)-MRI, extensively improves the sensitivity and specificity of the examination (9) and has attracted increasing attention from scholars as a developing tool for image characterization, treatment planning, and response assessment (10, 11). In recent years, mpMRI has created an exclusive opportunity to analyze complicated spatial image patterns through radiomics methods. Radiomics analysis can completely extract magnetic resonance (MR) image data, which contain information about the biological behavior of tumors. Advanced texture analysis of mpMRI provides more perceivable information than current clinical visual evaluations (10). It has been proven that radiomics have great potential for enhancing the clinical management of CC (12, 13).

However, to our knowledge, radiomics research on the predictive performance of LVSI by investigating patients with CC is relatively limited. Previous studies have mainly focused on conventional MRI (4, 14) or DCE-MRI quantitative parameter maps (15) and peritumoral information (16); small field-of-view (sFOV) HR-T2WI has not been considered. Since the conspicuity of a feature in an image largely depends on spatial resolution, better spatial resolution may lead to better diagnostic performance. Moreover, for some imaging tasks that involve high-frequency and high-contrast features in particular, the performance is predominantly determined by spatial resolution (17). We assume that sFOV HR-T2WI may provide further information to assess the disease status and enable more comprehensive CC phenotyping in strong radiomics signatures. A high-throughput screening feature vector acquired from sFOV HR-T2WI may enhance its diagnostic performance for identifying LVSI over other imaging modalities by reflecting bio-information, including lesion aggressiveness and vascularity. Therefore, in this study, we aimed to train and test a mpMRI-derived radiomics model, mainly based on sFOV HR-T2WI, to distinguish between CC with and without LVSI.

In this study, we proposed a novel radiomics-determined mathematical characterization of the CC risk phenotype. The model is primarily based on sFOV HR-T2WI and has a convincing predictive value. This suggests that the radiographic characteristics of mpMRI obtained *via* the standard-of-care represented by sFOV HR-T2WI might serve as a non-invasive LVSI identification method. The model may be useful for facilitating the risk stratification of patients in surgical procedures and neoadjuvant settings.

## Materials And Methods

### Patients

This retrospective study was approved by the Institutional Ethics Review Board, which waived the requirement for written informed patient consent. The study was conducted in accordance with the Declaration of Helsinki (18). The main cohort included 203 women with CC, confirmed by histopathological analysis after reviewing the institutional database of medical records from June 2012 to December 2018. Patients with distant metastases, history of prior radiotherapy or chemotherapy for CC, preceding or simultaneous malignant tumors, incomplete MRI sequences, and insufficient image quality were excluded (19). The final study population comprised 125 patients, and stratified sampling was adopted to assign them to the training and validation cohorts in an 8:2 ratio.

### MRI Protocol

All patients underwent MRI using a 3.0-T MRI scanner (Magnetom Skyra; Siemens Medical Solutions, Erlangen, Germany) with an 18-channel pelvic phased-array coil in the supine position. MRI was performed 10 days after a biopsy to avoid post-biopsy inflammation and 2 weeks before chemoradiation and surgery. Images were obtained from the pelvis to the renal hilum to detect the lymph node status. The set image protocol included sagittal turbo spin-echo (TSE)-T2WI and axial fat-suppressed (FS)-T2WI, axial DWI (9) (b values of 50 and 1000) with apparent diffusion coefficient (ADC) maps and sFOV high-resolution turbo T2-weighted sequences on the axial planes. Each patient was administered an injection of gadodiamide 0.2 mmol/kg (Ge Healthcare Shanghai Co., Ltd.). After 3 minutes, an FS-T1-weighted sequence (T1c) was acquired in the sagittal and axial planes (12). MRI datasets were retrieved from the picture achieving and communication system (PACS) for further image processing (20) (The MRI acquisition details are listed in **Supplementary Material, Table 1**).

### Image Preprocessing and Segmentation

To obtain robust features, linear interpolation was first adopted to resample the voxel size of the image to an isovolumetric voxel (1 × 1 × 1 mm^3^) before feature extraction (21). The Z-score method was used to standardize the image, and image intensity discretization was applied with a fixed bin width of 5 (22). Wavelet decomposition (wavelet transform, LLL, LLH, LHL, HLL, LHH, HLH, HHL, HHH) and Laplacian of Gaussian filters were applied to the image with sigma values of 3.0 and 5.0, respectively (23). An open-source software was subsequently used for medical image segmentation (ITK-SNAP, version 3.8.0; https://www.itksnap.org). The entire tumor area was assessed to avoid the presence of fluid in the cervical canal. The regions of interest were manually outlined by a single abdominal radiologist and confirmed by another abdominal radiologist (Y.C. and L.W., with 10 and 14 years of experience in pelvic radiological diagnosis, respectively) to reach a consensus regarding the CC tumor region (**Supplementary Material, Figures 1**, **2**). All patients were blinded to their clinical and histopathological characteristics. If the tumor region was not ascertained, the area was not included in the segmentation. After standardized pre-processing, 1037 radiomics features were extracted from the original and filtered images using PyRadiomics packages, including the shape features (14), first-order statistics (18), and texture features (including 24 gray-level co-occurrence matrix [GLCM] features, 16 gray-level run-length matrix [GLRLM] features, 16 gray-level size zone matrix [GLSZM] features, 14 gray level dependence matrix [GLDM] features, and 5 neighboring gray-tone difference matrix [NGTDM] features) (24). One month later, 30 patients were randomly selected for lesion definition and radiomics feature extraction by another radiologist (Y. J., with 14 years of experience in pelvic radiological diagnosis), who was blinded to the clinical and pathological findings. Robustness was assessed using the intra-class correlation coefficient (ICC).

### Radiomics Feature Extraction

It is imperative to select a useful and unique feature subset to avoid overfitting. The procedure for feature reduction was as follows: (a) Important features were obtained through a univariate analysis using the Mann-Whitney U test, in which *p*-values <0.01 were reserved. (b) The Spearman correlation coefficients for each pair of features were calculated, and features with a correlation coefficient > 0.9 were removed (25). (c) The least absolute shrinkage and selection operator (LASSO) was used on the remaining features, and the significant features with non-zero coefficients in the training cohort were selected to identify the LVSI status (11). (d) Stepwise regression of multiple factors was performed, retaining the feature set with the smallest Akaike’s information criterion (AIC) and then weighing a linear combination of selected features according to their respective coefficients (20, 26).

### Model Construction and Validation

The final hematoxylin and eosin (HE) stained pathological results were evaluated to determine the following factors: histology type, FIGO stage, presence of LVSI, and LNM. Multivariable logistic regression analysis was used for parameters including age, squamous cell carcinoma (SCC) antigen level, menopause, white blood cell count, neutrophil count, lymphocyte count, monocyte count, hemoglobin (HGB) level, blood platelet count, albumin level, neutrophil-to-lymphocyte ratio, platelet-to-lymphocyte ratio, and lymphocyte-to-monocyte ratio.

A clinical model for quantitative prediction of LVSI was established using the selected variates of HGB and SCC antigen to aid clinical practice (20). Logistic regression was used to establish the predictive models. Six independent models based on single MR sequences were constructed to estimate the validity of LVSI prediction, including ADC maps, sFOV HR-T2WI, axial FS-T2WI, sagittal T2WI, and axial and sagittal T1C models. The radiomics signature was used as an independent feature representing radiomics, along with clinical data. A radiomics model was then generated *via* a linear combination of the foremost features weighted by the corresponding coefficients of the aforementioned six sequences in the training set. Then, the Rad-score was computed for each and used in the validation set from the radiomics model. Clinical and radiomics models were constructed using logistic regression and followed AIC for backward feature selection (27).

In addition, we established a combined (COMB) model integrating the Rad-score with clinical predictors to test the added value of the LVSI differentiation model (23). To ensure a robust generalized performance of the models that best fit the observed data, 3-fold cross-validations were repeated 20 times in the training set. The performances of all models were then calculated for both the training and validation sets (20). All multivariable logistic regression formulas developed from the training sets were validated using the validation sets.

The calibration curves were computed to identify the consistency between the estimated probability of LVSI and the actual outcomes in both datasets (20).

### Clinical Practice/Decision Curve Analysis

To evaluate the incremental utility of the constructed classifiers, the decision curve of the radiomics model was plotted for both datasets (28). The net benefit was computed by subtracting the proportion of false-positive (FP) patients from the proportion of true-positive patients (TP), weighted by the relative harm of false-negative (FN) and false-positive results. For reference, the decision curves for treating all patients and treating no patients are shown. If the net benefit values of the model are greater than those of the two reference schemes, the model will show a clinical benefit (23). Through a threshold probability, the decision curve indicates which of the given models is the best for a patient or clinician (28).

### Statistical Analysis

Patient demographic data are presented as numbers (percentages) or medians (25th to 75th percentiles) for categorical and continuous variables (unless specified otherwise). Pearson’s χ2 or Fisher’s exact test was used to compare the categorical variables between groups, while the Mann-Whitney U test was used to compare the continuous variables (29). To select the features that allow for the identification of LVSI in patients with CC, multiple logistic regression models were fitted and compared using AIC (30). The diagnostic accuracy of optimal predictive parameters was determined using receiver operating characteristic (ROC) analysis, and the area under the ROC curve (AUC) was obtained (24). The model cutoff point was obtained using the Youden index, and the accuracy, sensitivity, and specificity were calculated (3). All statistical analyses were performed using R software (version 3.6.3, https://www.rproject.org), and a two-tailed *p*-value of <0.05, was considered statistically significant.

## Results

### Baseline Characteristics of Patients

A total of 125 patients were included in the study based on the inclusion and exclusion criteria. The training set included 100 patients with CC and a positive LVSI rate of 29.0%, while the validation set included 25 patients with CC and a positive LVSI rate of 20.0%. The median age of the patients in both groups was 48 years. The clinicopathological characteristics of the study population are shown in **Table 1**.

**Table 1:** Demographic and clinicopathological characteristics of patients.

Characteristic	Non-LVSI (n=91)	LVSI (n=34)	Total (n=125)	*P* value
Age (years)				0.803^1^
Mean (SD)	48.209 (9.317)	47.235 (8.235)	47.944 (9.014)	
Median (Q1, Q3)	47.000 (42.000, 55.000)	48.000 (42.250, 51.000)	47.000 (42.000, 54.000)	
Range	27.000 - 68.000	32.000 - 68.000	27.000 - 68.000	
SCC (ng/ml)				<0.001^1^
Mean (SD)	3.060 (7.394)	9.344 (14.503)	4.770 (10.176)	
Median (Q1, Q3)	1.200 (0.700, 2.950)	3.650 (1.050, 8.275)	1.400 (0.800, 3.500)	
Range	0.200 - 65.700	0.500 - 71.000	0.200 - 71.000	
Menopause				0.118^2^
Menopausal	32 (35.2%)	7 (20.6%)	39 (31.2%)	
Not menopausal	59 (64.8%)	27 (79.4%)	86 (68.8%)	
FIGO stage (%)				0.017^1^
I	49 (53.8%)	12 (35.3%)	61 (48.8%)	
II	38 (41.8%)	18 (52.9%)	56 (44.8%)	
III	4 (4.4%)	2 (5.9%)	6 (4.8%)	
IV	0 (0.0%)	2 (5.9%)	2 (1.6%)	
HistologyType				0.236^2^
non-squamous cell carcinoma	7 (7.7%)	5 (14.7%)	12 (9.6%)	
Squamous cell carcinoma	84 (92.3%)	29 (85.3%)	113 (90.4%)	
LNM (%)				0.011^2^
Non-metastasis	86 (94.5%)	27 (79.4%)	113 (90.4%)	
Metastasis	5 (5.5%)	7 (20.6%)	12 (9.6%)	
WBC (10-9/L)				0.343^1^
Mean (SD)	5.655 (1.746)	5.912 (1.623)	5.725 (1.711)	
Median (Q1, Q3)	5.300 (4.500, 6.500)	5.650 (4.700, 6.850)	5.300 (4.500, 6.700)	
Range	2.500 - 11.400	3.500 - 10.200	2.500 - 11.400	
NEUT (10-9/L)				0.720^1^
Mean (SD)	3.566 (1.422)	3.633 (1.331)	3.585 (1.393)	
Median (Q1, Q3)	3.300 (2.590, 4.215)	3.430 (2.700, 4.362)	3.380 (2.620, 4.340)	
Range	1.090 - 9.030	1.590 - 6.630	1.090 - 9.030	
LY (10-9/L)				0.088^1^
Mean (SD)	1.517 (0.497)	1.676 (0.538)	1.560 (0.511)	
Median (Q1, Q3)	1.470 (1.145, 1.860)	1.620 (1.373, 1.890)	1.510 (1.170, 1.870)	
Range	0.840 - 3.400	0.760 - 3.530	0.760 - 3.530	
MO (10-9/L)				0.334^1^
Mean (SD)	0.384 (0.121)	0.419 (0.155)	0.394 (0.132)	
Median (Q1, Q3)	0.360 (0.300, 0.480)	0.390 (0.300, 0.502)	0.380 (0.300, 0.480)	
Range	0.180 - 0.830	0.100 - 0.840	0.100 - 0.840	
HGB (g/L)				0.013^1^
Mean (SD)	125.121 (20.577)	114.471 (23.870)	122.224 (21.945)	
Median (Q1, Q3)	131.000 (113.000, 139.500)	120.500 (102.000, 131.750)	128.000 (108.000, 137.000)	
Range	56.000 - 163.000	58.000 - 152.000	56.000 - 163.000	
PLT (10-9/L)				0.402^1^
Mean (SD)	196.527 (71.603)	214.324 (87.256)	201.368 (76.227)	
Median (Q1, Q3)	202.000 (152.500, 243.500)	224.000 (154.250, 268.500)	202.000 (153.000, 250.000)	
Range	62.000 - 406.000	70.000 - 395.000	62.000 - 406.000	
ALB(g/L)				0.293^1^
Mean (SD)	43.043 (4.073)	42.024 (3.556)	42.766 (3.951)	
Median (Q1, Q3)	42.900 (41.000, 45.050)	42.450 (40.250, 44.325)	42.800 (40.700, 45.000)	
Range	33.600 - 62.000	34.800 - 47.700	33.600 - 62.000	
NLR				0.267^1^
Mean (SD)	2.515 (1.090)	2.386 (1.255)	2.480 (1.133)	
Median (Q1, Q3)	2.309 (1.691, 3.114)	2.005 (1.341, 2.919)	2.275 (1.619, 3.084)	
Range	0.846 - 6.076	0.958 - 6.196	0.846 - 6.196	
PLR				0.590^1^
Mean (SD)	138.569 (60.850)	142.783 (94.315)	139.715 (71.122)	
Median (Q1, Q3)	121.395 (98.924, 170.491)	119.531 (89.634, 161.903)	121.379 (98.755, 169.565)	
Range	22.615 - 315.116	44.759 - 519.737	22.615 - 519.737	
LMR				0.509^1^
Mean (SD)	0.267 (0.091)	0.266 (0.112)	0.267 (0.096)	
Median (Q1, Q3)	0.259 (0.203, 0.307)	0.234 (0.212, 0.298)	0.254 (0.205, 0.306)	
Range	0.114 - 0.697	0.049 - 0.607	0.049 - 0.697	

1. Mann-Whitney U test.

2. Pearson’s Chi-squared test.

LVSI, lymphovascular space invasion; SD, standard deviation; SCC, Squamous cell carcinoma antigen; FIGO, Federation International of Gynecology and Obstetrics stage; LNM, Lymph node metastasis; WBC, white blood cell; NEUT, neutrophil count; LY, lymphocyte count; MO, Monocyte count; HGB, Hemoglobin; PLT, blood platelet count; ALB, albumin; NLR, Neutrophil/lymphocyte ratio; PLR, Platelet/lymphocyte ratio; LMR, lymphocyte/monocyte ratio.

### Radiomics Feature Extraction and Selection

**Figure 1:**
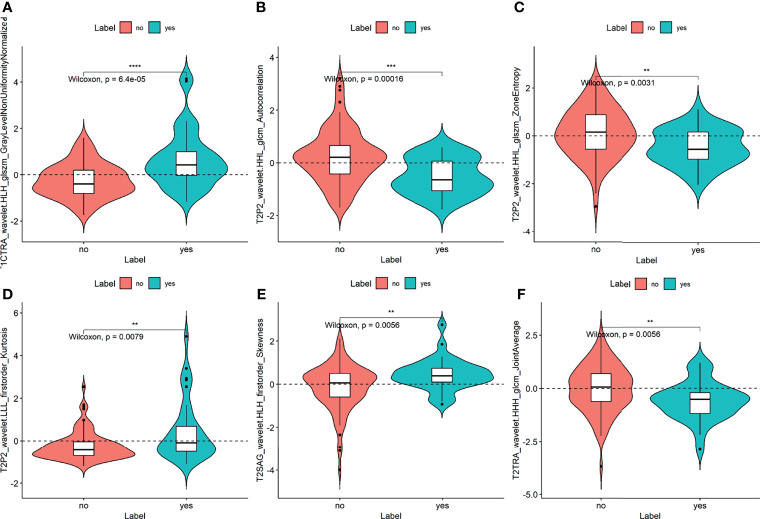
Plots **(A–F)** show the boxplots of the six radiomics features with a significant difference between the LVSI and non-LVSI subgroups in the training cohort. The symbol **, ***, **** means *P*-value < 0.01, 0.001, 0.0001, respectively.

A total of 1037 extracted features were further selected by univariate analysis, LASSO, and stepwise logistic regression analysis (**Supplementary Material Text 1**). Thirteen image features were reduced to only six potential independent predictors for the radiomics signature using the combination of all six single sequences. The most significant features were from the first-order and texture feature groups, including sFOV HR-T2WI (T2P2) _wavelet.LLL_firstorder_Kurtosis, T2P2_wavelet.HHL_glcm_Autocorrelation, T2P2_wavelet.HHL_glszm_ZoneEntropy, T1C transverse (TRA)_wavelet.HLH_glszm_GrayLevelNonUniformityNormalized, T2 sagittal (SAG)_wavelet.HLH_firstorder_Skewness, T2TRA_wavelet.HHH_glcm_JointAverage. They were all significantly different between the CC cases with and without LVSI (all *p* < 0.05; **Figure 1**). The results showed that the inter-observer ICC values of the radiomic features we used for the model were all > 0.8 (indicating good stability).

**Figure 2:**
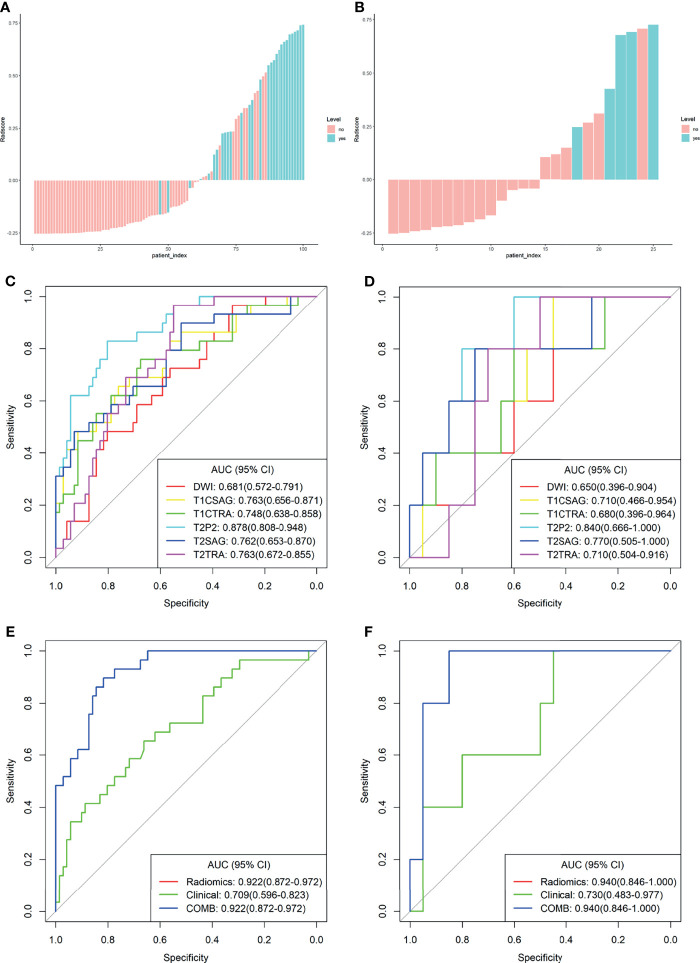
Plots **(A, B)** present the Rad-score in the training cohort **(A)** and the validation cohort **(B)** the red bars represent the scores for patients without LVSI, while the blue bars represent the scores for those with LVSI; plots **(C, D)** show the receiver operating characteristic (ROC) curves of the radiomics signature derived from single sequences in both sets; plots **(E, F)** present the ROC curves of the clinical model, radiomics model, and combined model.

All these features were included in the calculation formula for the Rad-score. The Rad-score distributions of each patient in the two cohorts are shown in **Figures 2** **A, B**.

Regarding clinical indicators, only SCC antigen and HGB independently represented the predictive variables for LVSI in patients with CC after the univariate and multivariate logistic regression analyses.

### Performance Comparison of Model Training and Validation

For comparison, the differentiation performance of the established models was quantified using the AUCs of the two groups. The proposed radiomics signatures distinguish LVSI status with AUCs ranging from 0.681 to 0.878. The AUC values for sFOV HR-T2WI, ADC map, axial FS-T2WI, sagittal T2WI, axial T1c, and sagittal T1c were 0.878, 0.681, 0.763, 0.762, 0.748, and 0.763 in the training cohort (**Figure 2C**). Regarding the LVSI classification method, the AUC of the sFOV HR-T2WI-based model was significantly higher than that of the other single-layer models (**Figures 2C, D** **)**.

The calculated Rad-score was used to construct the corresponding radiomics model. Compared with a single sequence model, the radiomics model provided an even better predictive model for LVSI, yielding an AUC of 0.922 and 0.940, a sensitivity of 0.897, 1.000, and specificity of 0.817, 0.700, respectively (**Table 2**).

**Table 2:** Discriminative value of each parameter in differentiating LVSI.

Method	AUC (95%CI)	accuracy	sensitivity	specificity	PPV	NPV	FP	FN
DWI	0.681 (0.572-0.791)	0.510	0.966	0.324	0.368	0.958	0.676	0.034
T1C SAG	0.763 (0.656-0.871)	0.730	0.655	0.761	0.528	0.844	0.239	0.345
T1C TRA	0.748 (0.638-0.858)	0.700	0.759	0.676	0.489	0.873	0.324	0.241
T2P2	0.878 (0.808-0.948)	0.810	0.828	0.803	0.632	0.919	0.197	0.172
T2 SAG	0.762 (0.653-0.870)	0.630	0.897	0.521	0.433	0.925	0.479	0.103
T2 TRA	0.763 (0.672-0.855)	0.670	0.966	0.549	0.467	0.975	0.451	0.034
Radiomics	0.922 (0.872-0.972)	0.840	0.897	0.817	0.667	0.951	0.183	0.103
Clinical	0.709 (0.596-0.823)	0.660	0.655	0.662	0.442	0.825	0.338	0.345
COMB	0.922 (0.872-0.972)	0.840	0.897	0.817	0.667	0.951	0.183	0.103

AUC, area under ROC curve; CI, confidence interval; PPV, positive predictive value; NPV, negative predictive value; DWI, ADC map model; TIC SAG, sagittal T1C model; TIC TRA, axial T1c model; T2P2, sFOV HR-T2WI model; T2 SAG, sagittal T2WI model; T2 TRA, axial T2WI model; COMB, combined model; FP, False Positive; FN, False Negative.

The clinical model was developed using the selected variates, showing the degree of predictive performance for LVSI and achieving an AUC of 0.709 (95% CI, 0.596-0.823) and 0.730 (95% CI, 0.483-0.977) in the training and validation sets, respectively (**Table 2** and **Figures 2E, F** **)**. To ensure the accuracy of the COMB model, the clinical factors were not included. Thus, the proposed model was constructed based on only the radiomic model, and both had the same performance.

**Figure 3:**
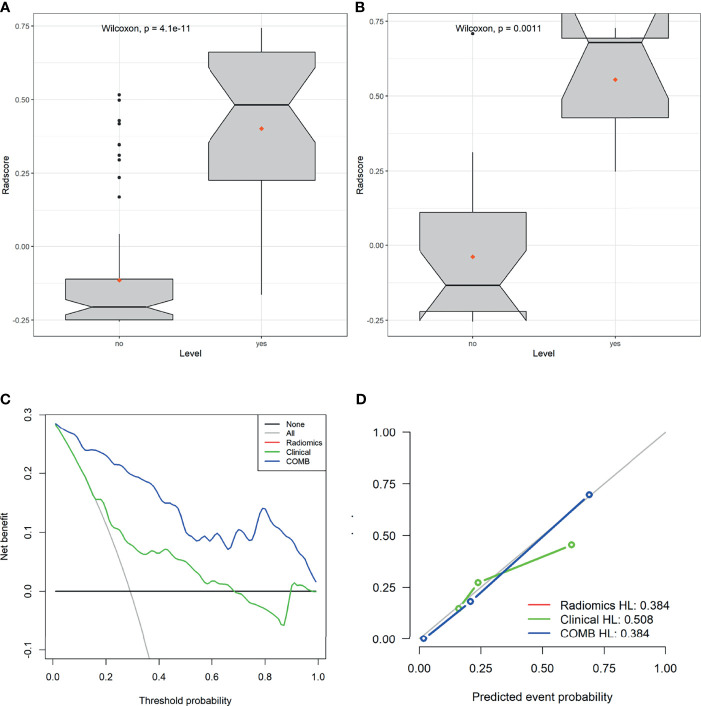
Plots **(A, B)** show the boxplots of the Rad-score in both cohorts, respectively. **(C)** Decision curve analysis for the radiomics signature in the training set. The Y-axis shows the net benefit; the X-axis shows the threshold probability. The decision curves showed that if the threshold probability falls in the range of 5%-95%, the radiomics model achieves the best clinical benefit than other models. **(D)** The calibration curve showed that the predicted LVSI was very close to the actual value.

The derived Rad-score was significantly higher in patients with LVSI than in those without LVSI in both groups (*p <*0.05) (**Figures 3A, B** **)**. The multiple regression analysis integrating all variables demonstrated a statistically significant difference between CC with and without LVSI. All models performed well in LVSI identification. Comparative analysis of the randomly drawn 3-fold cross-validation sets showed low variability of results, indicating a stable prediction performance (**Supplementary Material, Figure 3**). Similar results were also observed between the training and validation sets. No statistically significant differences were observed in most of the constructed models, showing the strong robustness of the models, including the clinical, single, radiomics, and COMB models of the two groups (**Figures 2C** **–F**). The radiomics characterization developed the LVSI identification to a significantly higher performance standard. The predictive capacity (sensitivity, specificity, accuracy, FP, and FN) of all models used to identify LVSI in the training cohort are listed in **Table 2**.

### Clinical Usefulness

The decision curve analysis (DCA) of the radiomics, clinical, and COMB models is presented in **Figure 3C**. The decision curves showed that the COMB model added more benefits than the other models and simple strategies, such as clinical data. The calibration curve of the radiomics model showed good agreement between prediction and observation in the training set (**Figure 3D**).

## Discussion

In this study, we trained and validated a united model incorporating the mpMRI radiomics signature for individualized LVSI prediction in patients with CC before surgery. The proposed radiomics signature, especially the model based on sFOV HR-T2WI, showed good differentiation ability between the two groups, outperforming the clinical data and other single models. Additionally, multiple radiomics signatures were integrated into a combined model, significantly enhancing the accuracy and model-fitting degrees. The classifier also achieved better performance in the non-LVSI subgroup than in the LVSI subgroup. Hence, we suppose that mpMRIs coupled with radiomics methods may be an effective tool in clinical decision-making regarding image-based differentiation between the presence and absence of LVSI.

The 5-year overall survival rate of patients without LVSI is higher than that of patients with LVSI (3). Therefore, accurate preoperative prediction of LVSI status is of great importance in guiding individualized treatment strategies for patients with CC (6). However, visual evaluation cannot ensure that the characteristic differences are identified in the clinical setting. It is essential to develop a non-invasive, and highly efficient predictive approach for LVSI identification. Radiomics supposes that intralesional heterogeneity is difficult to observe with the naked eye, but it can be detected using the spatial distribution of voxel intensities (23). Imperceptible tumor information can be revealed by radiomics analysis, which will gradually help overcome the limitations of pure visual image interpretation. Radiomics has shown the potential to predict LVSI in endometrial carcinoma (31), breast cancer (32), and gastric cancer (33). Image features may also provide insight into the nature of CCs beyond the scope of visual assessment (34). In this study, we used an automatic high-throughput screening feature extraction method to obtain 1037 radiomics features using mpMRI, which comprehensively reflects the imaging phenotype of CC.

The radiomics model constructed in this study exhibited favorable discrimination in both the training and validation sets, with an AUC of 0.922, 0.940, respectively. Several studies have evaluated the feasibility of using MRI for visual or computerized analyses of LVSI identification. The predictive capacity of our model was comparable to that of models reported in previous studies. The Rad-score obtained from Positron Emission Tomography-Computed Tomography (PET/CT) images with molecular proteins is closely related to that in cases of early CC with LVSI (35). In previous studies, the AUC of the support vector machine constructed using only conventional T2WI reached 0.7356 (14), while the radiomics nomogram developed on T1c images relevant to LVSI had an AUC of 0.754 (4). Our study confirmed that the Rad-scores of T2WI, T1c, and ADC maps of patients with LVSI were significantly higher than those of patients without LVSI. The heterogeneity or textual pattern complexity of the features observed in cases with LVSI was higher than in those without LVSI. A further study has investigated the potential of radiomics and deep learning fusion strategy, showing that the features derived from the region with a radial dilation distance of 8 mm and 4 mm outside the tumor achieved the best classification ability for T1c and T2WI, respectively (16). In a small pilot study of 56 patients, multi-sequence-based radiomics signatures showed great differentiation value for LVSI, especially the functional maps obtained from DCE-MRI, with AUCs ranging from 0.659 to 0.814 (15). Although the patient sample size was small and there were no external test sets, the study indicated the prime potential of quantitative image feature correlation with LVSI. The use of DCE sequences may reflect the cell structure and microangiogenesis of tumor tissue. The derived quantitative parametric maps may also contain more tumor hemodynamic information, adding useful diagnostic information to the radiomics methods. However, the generalizability of existing results dependent on visual evaluation is limited by the complicated quantitative image acquisition process and markers, even for experienced observers. In addition, technical differences between the quantitative sequences or the absence of external validation limit their clinical application value. Our findings accord with and support the current trend of reducing research time and costs by omitting DCE quantitative maps from the conventional MRI protocol and then using routine scanning sequences. The routinely obtained sFOV HR-T2WI sequence is also less expensive and time-saving than contrast-enhanced MRI and DWI. Through further validation, this approach may serve as a complementary imaging method for pelvic MRI in patients with CC.

Although the image data were pre-processed before feature extraction, such as isotropic resampling of the voxel size post-acquisition, it might not eliminate the initial resolution differences completely. We observed some distinctions between images obtained with different spatial resolutions. The sFOV HR-T2WI acquired more robust features than normal images. Previously, Mayerhoefer systematically studied the influence of image interpolation and matrix size of imaging features, indicating that image data acquisition at higher resolution achieves better diagnostic efficiency (36). A phantom study also demonstrated that image spatial resolution is of great significance for the robustness and reproducibility of MRI radiomics (37). Among the features from MRI sequences, those from sFOV HR T2WI accounted for the highest proportion, suggesting the importance of sFOV HR-T2WI-derived radiomics signatures in this classification model. A reasonable interpretation could be summarized as follows: HR-MRI based on sFOV sequences allow for artifact reduction and smooth fusion with morphologic T2WI; combining an HR-MRI and sFOV readout may contribute to increasing the accuracy of diagnosis (38). sFOV HR-MRI is characterized by excellent spatial resolution, high image complexity, and large intra-class differences, and provides high-contrast structural and functional information, including a large amount of spatial information about the tumor, rich shape, texture, structure, and neighborhood relationship features. In addition, sFOV HR-MRI could explicitly detect the tumor microenvironment, capture the quantifiable differences occurring in the tissue vasculature, and enrich existing imaging features. For these reasons, sFOV HR-T2WI can provide abundant visual information for LVSI detection. The results further reveal the importance of spatial resolution in radiomics measures. Frequency-selective axial FS-T2WI differs from sFOV HR T2WI. It is unclear whether there is a difference in highlighting the histopathological features of CC in T2WI with or without fat suppression. Other tissue components, such as blood and protein, that have the same T1 time as the fat in the lesion may be inhibited when fat signaling is suppressed. Some image features that characterize subtle changes in the vasculature of CC may also be weakened, in which some microscopic changes may be closely related to LVSI. A similar result was observed in a previous study, wherein T2 was slightly higher than FS-T2WI for LVSI prediction in patients with CC (AUC= 0.710, 0.697) (15). The ADC models show the worst performance probably due to poor spatial resolution and limited signal-to-noise ratio. Some imaging features may be weakened owing to partial volume effects, resulting in relatively minor differences in the feature value (15, 39, 40). This, in turn, reduces the machine’s ability to reveal subtle, imperceptible, local structural differences in tumor components. This may be a key reference for the machines to identify lesions. These findings are consistent with those of several previous studies (15, 39), and those associated with endometrial carcinoma (40). We hypothesized that functional and anatomical images have unique advantages in characterizing lesion heterogeneity. LVSI is a pathological finding, that may be more inclined to be an anatomical, and morphological characteristic. Therefore, anatomical images may be more helpful for LVSI observation, while ADC maps may not have significant advantages in characterizing the vascular tumor cells in CC.

To perform a multi-resolution analysis of the imaging data and display the image details at different levels, we decomposed the original image through a discrete, first-order, wavelet transformation, Gaussian filter (41). To further train the radiomics signature, the candidate imaging features were reduced to only six important and robust parameters. The absolute values of the coefficients were calculated using the LASSO algorithm, and the selected non-zero coefficient features were analyzed to reflect the contributions of relevant features to tumor risk stratification (42). Wavelet features (6/6) account for all those used in our optimal radiomics signature, implying a closer relationship with the LVSI status. Wavelet transformation can split image data into different frequency components using a three-dimensional (3D) analytical approach (42). The wavelet features may reflect multi-frequency information indistinguishable to the naked eye on multiple scales to lucubrate the spatial heterogeneity of tumors (23), which may also explain why radiologists cannot predict LVSI by visually examining MR images. In this study, all high-dimensional features in the LVSI subgroup were remarkably higher than those in the non-LVSI subgroup. Several MRI-based radiomics studies have confirmed that wavelet transformation is a significant component of radiomics signature construction (23, 41). Thus, using texture features and higher-order statistics may considerably characterize the tumor heterogeneity in CCs.

False-positive results could lead to surgery and needless concurrent chemoradiation therapy (CCRT) as the first treatment choice, accompanied by adjuvant chemoradiotherapy and more serious complications. However, false-negative LVSI status may cause undertreatment of aggressive squamous cell carcinomas. Both results should be avoided as much as possible. Profiting by the relatively high sensitivity of our radiomics signature, 31 of 34 patients with histopathological LVSI were correctly identified. The model can reduce the misdiagnosis rate (FP=0.183) and missed diagnosis rate (FN=0.103). We examined the images that were not picked up. Three tumors grew to a large size and showed significantly larger necrotic foci. They were invasive CC that had invaded the uterus and stroma of the endometrium. We assume that some textural features of these tumor tissues might not have been objectively captured by radiomic measurements because they were affected by the mixing of the mucous membrane, mucus, and extensive necrosis.

This study also indicated that the radiomics model established by incorporating six independent variates was superior to all single imaging techniques, which showed strong calibration and differentiation ability in data groups, perhaps the complementary value of these data. Images obtained from multiple scanning sequences usually reflect different aspects of a lesion, including tumor intensity, cellularity, and vascularization (23). This implies that it may have some “significant” additional value compared to the single-slice analysis. The features observed from different scanning directions showed no differences in diagnostic performance. Combining these sequences, such as the combination of sagittal and axial plane images, could maximize their respective values and reflect more comprehensive tumor information. We suppose that volumetric analysis may comprehensively reflect heterogeneity within the tumor and is not affected by the scan plane. In contrast, 3D images contain higher dimensions of detailed information and describe the outer superficial information of the tumor tissue, which is theoretically more aggressive (41). The radiomics model greatly improved the application of clinical characteristics, while the COMB model integrating clinical variates showed no significant improvement in accuracy. This may be attributed to the weak correlation between the LVSI and clinical factors. Although the relationship between chronic blood loss parameters, such as erythrocyte count, has also been investigated (4). According to our results, the contribution of clinical data to the prediction model was insufficient to improve the predictive accuracy. The combined model realized visualization, and individualized prediction of LVSI also showed higher diagnosability and more net benefits. The threshold probability of the DCA almost precedes that of the single-layer models (42), suggesting that integrating mpMRI radiomics data enables the best models. The radiomics model had the highest net benefit within a reasonable threshold probability range, indicating its incremental value in terms of clinical application. The results exhibited an impressive predictive capacity for the radiomics signature. Because the composition ratio (i.e., the ratio of CC without LVSI to CC with LVSI) was comparable in both cohorts, the combined clinical-radiomics model proposed here is reliable and shows the potential to guide clinical practice. The classifiers were trained using the 3-fold cross-validation method, suggesting that the proposed model achieved a higher and stable diagnostic performance. Radiomics is likely to provide sufficient information to promote risk stratification, prognostic prediction, and treatment.

This study has some limitations. First, this retrospective study did not review external validation datasets. The validity of our findings must be interpreted with caution because the results were established in a single center with limited sample size. Selection bias was inevitable. The databases of cohorts for external validation are being developed in an ongoing study, including one dataset from our institution sampled during a later period (2019–2021) and the two datasets from additional external institutions. Second, manual segmentation of the VOI is a labor-intensive and time-consuming process. Developing a reliable tool for the automatic segmentation and computation of radiomic signatures is crucial for promoting the feasibility of radiometric measures. Third, because of limited positive cases and classification of subtypes that cannot be provided by HE staining results, we did not compare the LVSI subgroups and did not explore the imaging differences between blood vascular invasion and 1ymph vascular invasion in-depth. Fourth, the genomic characteristics were not incorporated into the model. However, this subject requires further investigation. To better generalize our results, it is necessary to overcome these limitations and validate the published data.

Despite several limitations, our study is a proof of the concept that standardly acquired sFOV HR-T2WI can reliably characterize risk stratification and guide further studies to explore individual therapies using MRI radiomics for patients with CC. External validation and prospective studies are required to verify our findings.

On current study suggests that sFOV HR-T2WI-based radiomics provides a precise estimation of CC aggressiveness. Combining the mpMRI-based radiomics signature could support clinical decision-making. Further investigations are warranted to evaluate the actual potential of radiomics to help discriminate the LVSI status in CC.

## Data Availability Statement

The original contributions presented in the study are included in the article/**Supplementary Material**. Further inquiries can be directed to the corresponding author.

## Ethics Statement

The studies involving human participants were reviewed and approved by The Ethics Committee of Gansu Provincial Hospital. Written informed consent for participation was not required for this study in accordance with the national legislation and the institutional requirements.

## Author Contributions

YC, GH, and LZ conception and design. GH, PW, and LW: development of methodology (acquired and managed patients, provided facilities, etc.). YJ, JR, and YM: analysis and interpretation of data (e.g., statistical analysis, biostatistics, and computational analysis). YC, XM, and JR: writing, review, and/or revision of the manuscript. PW, LW, and YJ: administrative, technical, or material support (i.e., reporting or organizing data, constructing databases). All authors contributed to the article and approved the submitted version.

## Funding

This study was supported by the Project of Gansu Provincial Health Commission (No. GSWSKY2020-15) and a grant from the Gansu Provincial Hospital of China (No.20GSSY1-18).

## Conflict of Interest

Author JR was employed by GE Healthcare China.

The remaining authors declare that the research was conducted in the absence of any commercial or financial relationships that could be construed as a potential conflict of interest.

The reviewer PP declared a shared affiliation with one of the authors, JR, to the handling editor at time of review.

## Publisher’s Note

All claims expressed in this article are solely those of the authors and do not necessarily represent those of their affiliated organizations, or those of the publisher, the editors and the reviewers. Any product that may be evaluated in this article, or claim that may be made by its manufacturer, is not guaranteed or endorsed by the publisher.

## Abbreviations

CC, Cervical cancer; LVSI, Lymphovascular space invasion; LNM, Lymph node metastasis; FIGO, Federation International of Gynecology and Obstetrics; mpMRI, Multi-parametric magnetic resonance imaging; VOI, Volume of interest; HR-T2WI, High-resolution T2-weighted MRI; T2WI, T2-weighted MRI; DWI, diffusion-weighted imaging; DCE, Dynamic Contrast Enhancement; MR, magnetic resonance; sFOV, Small field of view; TSE, turbo spin-echo; FS, fat- suppressed; ADC, Apparent diffusion coefficient; T1c, Contrast-enhanced T1-weighted MRI; PACS, picture achieving and communication system; GLCM, Gray-level Co-occurrence Matrix; GLRLM, Gray-level Run-length Matrix; GLSZM, Gray-level Size Zone Matrix; GLDM, Gray Level Dependence Matrix; NGTDM, Neighbouring Gray Tone Difference Matrix; ICC, Intra-class correlation coefficient; LASSO, Least absolute shrinkage and selection operator; AIC, Akaike’s information criterion; HE stained, hematoxylin and eosin (HE) stained; SCC, Squamous cell carcinoma antigen; HGB, Hemoglobin; COMB, combined; FP, False Positive; TP, positive patients; FN, False Negative; ROC curve, Receiver operating characteristic curve; AUC, Area under receiver operating characteristic curve; WBC, White blood cell; NEUT, Neutrophil count; LY, Lymphocyte count; MO, Monocyte count; PLT, Platelet count; ALB, Albumin; NLR, neutrophil-to-lymphocyte ratio; PLR, platelet-to-lymphocyte ratio; LMR, lymphocyte-to-monocyte ratio; T2P2, sFOV HR-T2WI; TRA, transverse; SAG, sagittal; Cis, Confidence intervals; PPV, positive predictive value; NPV, negative predictive value; DCA, Decision curve analysis; PET/CT, Positron Emission Tomography-Computed Tomography; 3D, Three-dimensional; CCRT, concurrent chemoradiation therapy.
