# Supplementary material for: Multi-Parametric Magnetic Resonance Imaging-Based Radiomics Analysis of Cervical Cancer for Preoperative Prediction of Lymphovascular Space Invasion
Source: Front Oncol. 2022 Jan 12;11:663370. doi: 10.3389/fonc.2021.663370 (PMC8790703; doi:10.3389/fonc.2021.663370)
Supplement: Supplementary file 1 [file DataSheet_1.docx]

| Sequence | Slice orientation |  | Repetition time (ms) | Echo time (ms) | Field of view | Acquisition matrix | Flip Angle(°) | Bandwidth(HZ) | Slice thickness | fat suppression |
| --- | --- | --- | --- | --- | --- | --- | --- | --- | --- | --- |
| T2W_P2 | TRA | FLASH | 7500 | 101 | 200×200 | 320×320 | 160 | 200 | 3.5/ | - |
| T2WI | TRA | FLASH | 7451 | 85 | 320×320 | 384×269 | 180 | 169 | 5 | + |
| T2WI | SAG | FLASH | 3000 | 86 | 260×260 | 320×256 | 140 | 206 | 6 | - |
| T1c | TRA | VIBE | 3.23 | 1.22 | 380×309 | 320×240 | 9 | 520 | 1.2 | + |
| T1c | SAG | VIBE | 3.10 | 1.25 | 350×262 | 384×269 | 9 | 690 | 3 | + |
| DWI | TRA | EPI | 6500 | 74 | 380×380 | 192×163 | - | 1532 | 5 | + |

**Supplementary Materials**

TABLE 1｜mpMRI scan protocols

T2W_P2, Small field of view High-resolution T2-weighted imaging; T1c, Contrast-enhanced T1-weighted, DWI, Diffusion weighted imaging; TRA and SAG represent transverse view and sagittal view respectively.

**FIGURE 1**｜A 56-year-old CC woman with LVSI. Plots (**A-F**) show the regions of interest (ROIs) in small field-of-view high-resolution T2-weighted MRI (sFOV HR-T2WI), axial fat-suppressed (FS)-T2WI, axial contrast-enhanced T1-weighted MRI (T1c), apparent diffusion coefficient (ADC), sagittal T2WI, sagittal T1c, respectively.


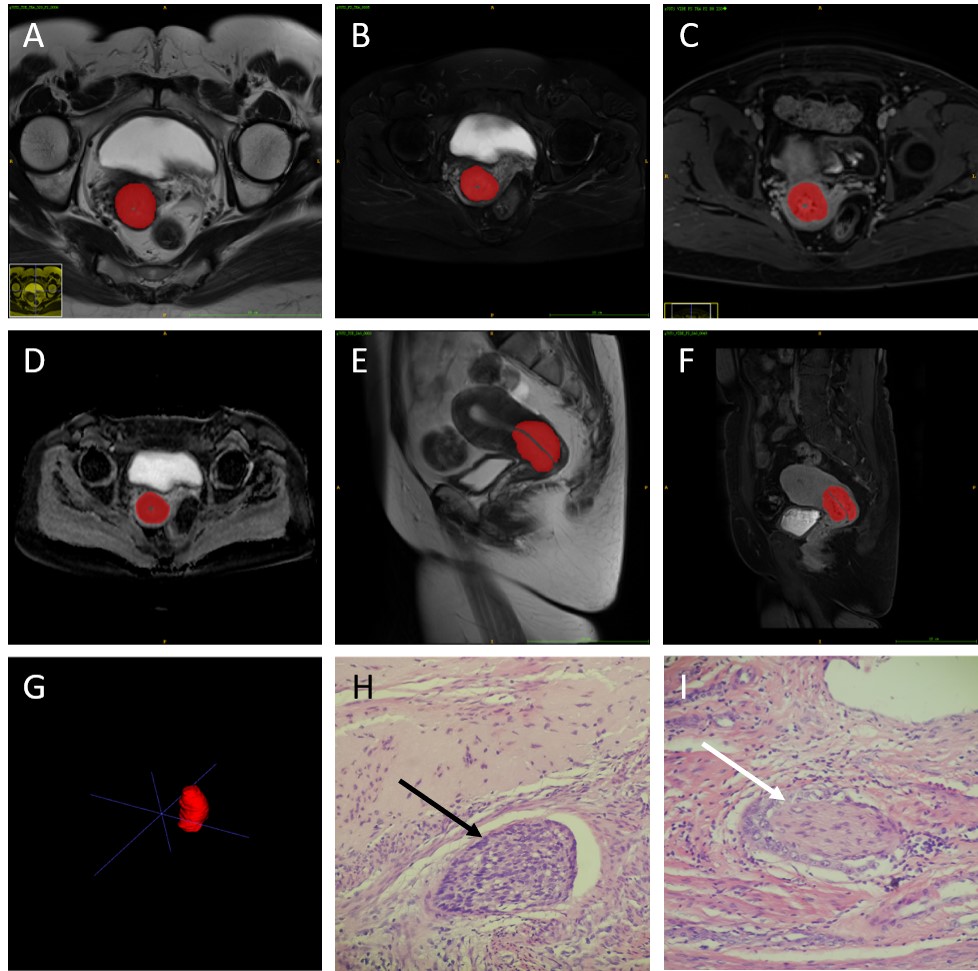


**FIGURE 2**｜A 51-year-old CC woman without LVSI. Plots (**A-F**) show the ROI in sFOV HR-T2WI, axial FS-T2WI, axial T1c, ADC, sagittal T2WI, sagittal T1c, respectively.


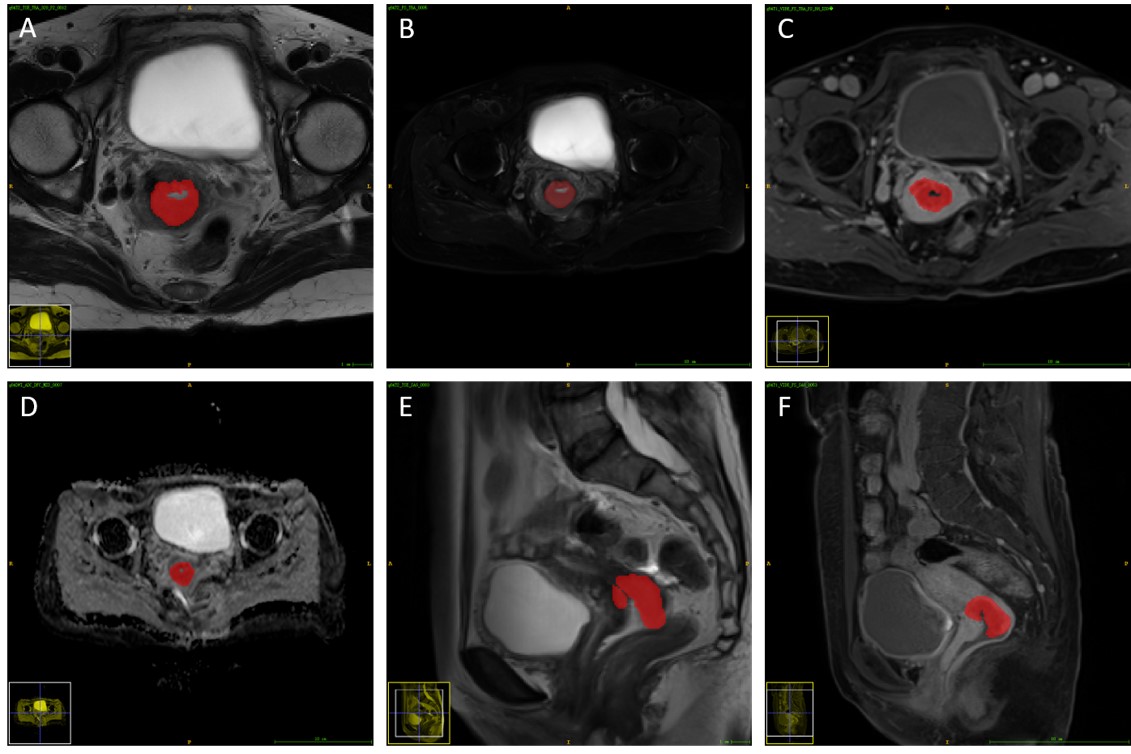


**FIGURE 3**｜Box plot shows distribution of results for 3-fold cross-validation performed 20 times.


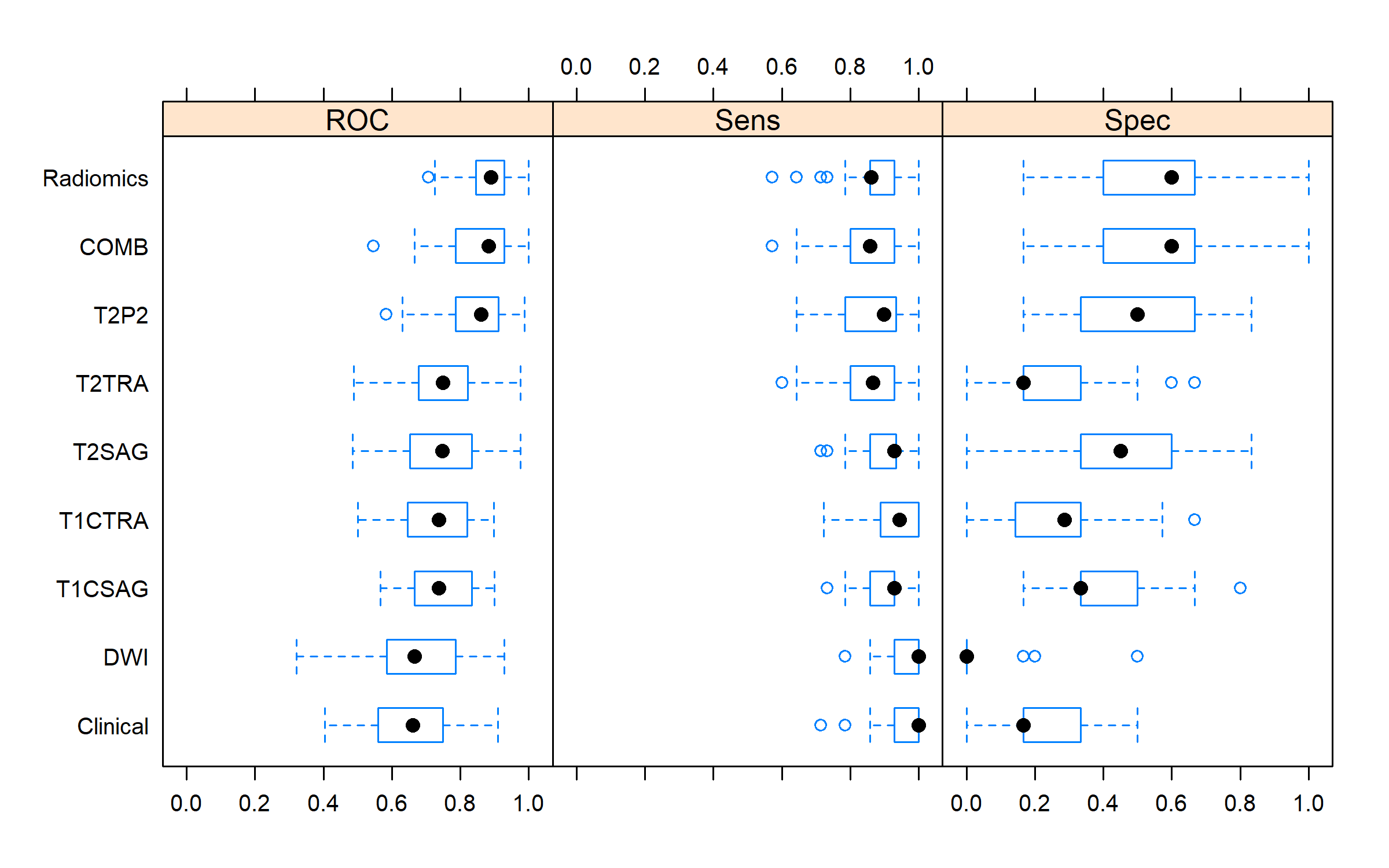


**Text 1**

**The Radiomic feature sets are as follows:**

1. shape features (14)

Elongation, Flatness, LeastAxisLength, MajorAxisLength, Maximum2DDiameterColumn, Maximum2DDiameterRow, Maximum2DDiameterSlice, Maximum3DDiameter, MeshVolume, MinorAxisLength, Sphericity, SurfaceArea, SurfaceVolumeRatio, VoxelVolume

1. first-order grey-level statistics features (18)

10Percentile, 90Percentile, Energy, Entropy, InterquartileRange, Kurtosis, Maximum, MeanAbsoluteDeviation, Mean, Median, Minimum, Range, RobustMeanAbsoluteDeviation, RootMeanSquared, Skewness, TotalEnergy, Uniformity, Variance

1. textural features (75)
2. grey-level co-occurrence matrices (GLCM, 24 features)

glcm_Autocorrelation, glcm_ClusterProminence, glcm_ClusterShade, glcm_ClusterTendency, glcm_Contrast, glcm_Correlation, glcm_DifferenceAverage, glcm_DifferenceEntropy, glcm_DifferenceVariance, glcm_Id, glcm_Idm, glcm_Idmn, glcm_Idn, glcm_Imc1, glcm_Imc2, glcm_InverseVariance, glcm_JointAverage, glcm_JointEnergy, glcm_JointEntropy, glcm_MCC, glcm_MaximumProbability, glcm_SumAverage, glcm_SumEntropy, glcm_SumSquares

1. grey-level run length matrices (GLRLM, 16 features)

glrlm_GrayLevelNonUniformity, glrlm_GrayLevelNonUniformityNormalized, glrlm_GrayLevelVariance, glrlm_HighGrayLevelRunEmphasis, glrlm_LongRunEmphasis, glrlm_LongRunHighGrayLevelEmphasis, glrlm_LongRunLowGrayLevelEmphasis, glrlm_LowGrayLevelRunEmphasis, glrlm_RunEntropy, glrlm_RunLengthNonUniformity, glrlm_RunLengthNonUniformityNormalized, glrlm_RunPercentage, glrlm_RunVariance, glrlm_ShortRunEmphasis, ShortRunHighGrayLevelEmphasis, glrlm_ShortRunLowGrayLevelEmphasis,

1. grey-level size-zone matrices (GLSZM, 16 features)

glszm_GrayLevelNonUniformity, glszm_GrayLevelNonUniformityNormalized, glszm_GrayLevelVariance, glszm_HighGrayLevelZoneEmphasis, glszm_LargeAreaEmphasis, glszm_LargeAreaHighGrayLevelEmphasis, glszm_LargeAreaLowGrayLevelEmphasis, glszm_LowGrayLevelZoneEmphasis, glszm_SizeZoneNonUniformity, glszm_SizeZoneNonUniformityNormalized, glszm_SmallAreaEmphasis, glszm_SmallAreaHighGrayLevelEmphasis, glszm_SmallAreaLowGrayLevelEmphasis, glszm_ZoneEntropy, glszm_ZonePercentage, glszm_ZoneVariance,

1. gray level dependence matrices (GLDM, 14 features)

gldm_DependenceEntropy, gldm_DependenceNonUniformity, gldm_DependenceNonUniformityNormalized, gldm_DependenceVariance, gldm_GrayLevelNonUniformity, gldm_GrayLevelVariance, gldm_HighGrayLevelEmphasis, gldm_LargeDependenceEmphasis, gldm_LargeDependenceHighGrayLevelEmphasis, gldm_LargeDependenceLowGrayLevelEmphasis, gldm_LowGrayLevelEmphasis, gldm_SmallDependenceEmphasis, gldm_SmallDependenceHighGrayLevelEmphasis, gldm_SmallDependenceLowGrayLevelEmphasis

1. neighborhood grey-tone difference matrices (NGTDM, 5 features)

ngtdm_Busyness, ngtdm_Coarseness, ngtdm_Complexity, ngtdm_Contrast, ngtdm_Strength
